# Toxin-Producing Endosymbionts Shield Pathogenic Fungus against Micropredators

**DOI:** 10.1128/mbio.01440-22

**Published:** 2022-08-25

**Authors:** Ingrid Richter, Silvia Radosa, Zoltán Cseresnyés, Iuliia Ferling, Hannah Büttner, Sarah P. Niehs, Ruman Gerst, Kirstin Scherlach, Marc Thilo Figge, Falk Hillmann, Christian Hertweck

**Affiliations:** a Department of Biomolecular Chemistry, Leibniz Institute for Natural Product Research and Infection Biology (HKI), Jena, Germany; b Junior Research Group Evolution of Microbial Interactions, Leibniz Institute for Natural Product Research and Infection Biology (HKI), Jena, Germany; c Research Group Applied Systems Biology, Leibniz Institute for Natural Product Research and Infection Biology (HKI), Jena, Germany; d Institute of Microbiology, Faculty of Biological Sciences, Friedrich Schiller University Jena, Jena, Germany; e Faculty of Biological Sciences, Friedrich Schiller University Jena, Jena, Germany; Max Planck Institute for Marine Microbiology

**Keywords:** microbial interactions, natural products, rhizoxin, *Rhizopus*, symbiosis, microbial ecology, secondary metabolism

## Abstract

The fungus Rhizopus microsporus harbors a bacterial endosymbiont (*Mycetohabitans rhizoxinica*) for the production of the antimitotic toxin rhizoxin. Although rhizoxin is the causative agent of rice seedling blight, the toxinogenic bacterial-fungal alliance is, not restricted to the plant disease. It has been detected in numerous environmental isolates from geographically distinct sites covering all five continents, thus raising questions regarding the ecological role of rhizoxin beyond rice seedling blight. Here, we show that rhizoxin serves the fungal host in fending off protozoan and metazoan predators. Fluorescence microscopy and coculture experiments with the fungivorous amoeba *Protostelium aurantium* revealed that ingestion of R. microsporus spores is toxic to *P. aurantium*. This amoebicidal effect is caused by the dominant bacterial rhizoxin congener rhizoxin S2, which is also lethal toward the model nematode Caenorhabditis elegans. By combining stereomicroscopy, automated image analysis, and quantification of nematode movement, we show that the fungivorous nematode Aphelenchus avenae actively feeds on R. microsporus that is lacking endosymbionts, whereas worms coincubated with symbiotic R. microsporus are significantly less lively. This study uncovers an unexpected ecological role of rhizoxin as shield against micropredators. This finding suggests that predators may function as an evolutionary driving force to maintain toxin-producing endosymbionts in nonpathogenic fungi.

## INTRODUCTION

**FIG 1:**
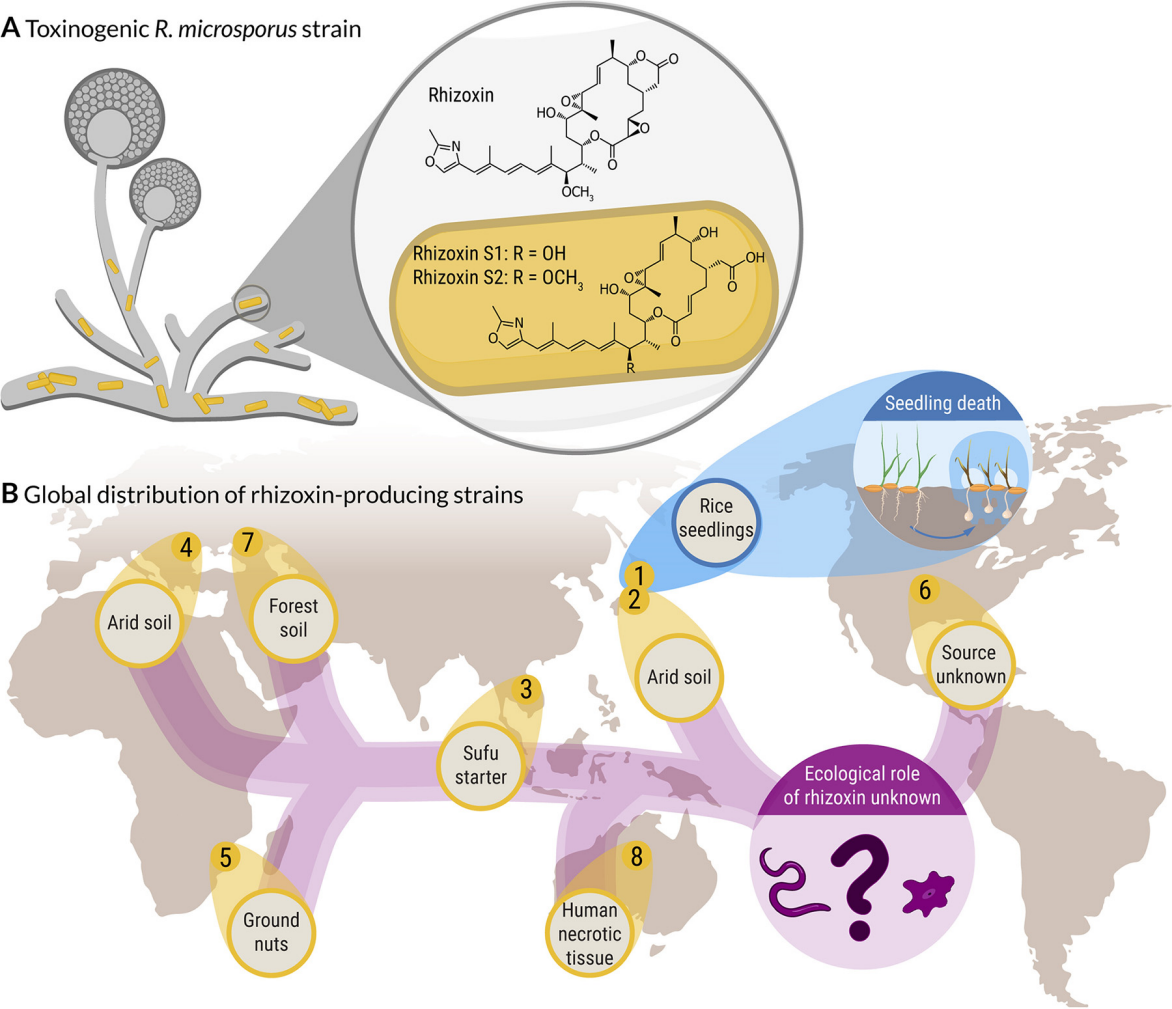
Global distribution of a toxin-producing bacterial-fungal symbiosis. (A) Symbiotic bacteria (*Mycetohabitans* sp.) residing within the fungal hypha of R. microsporus, produce a mixture of toxic secondary metabolites (rhizoxins). (B) Rhizoxin-producing *Rhizopus*-*Mycetohabitans* strains were isolated from environmental samples from geographically distinct sites covering all five continents. In one of the eight toxinogenic strains (R. microsporus ATCC 62417, blue), rhizoxin causes blight disease in rice seedlings, while the ecological role of rhizoxin in the other, nonpathogenic *Rhizopus* strains is currently unknown.

The filamentous fungus Rhizopus microsporus (phylum Mucoromycota) plays an important role in a variety of fields, including agriculture, biotechnology, and medicine. While some strains are being used for food fermentation and metabolite production others cause mucormycosis in immunocompromised patients (1, 2). However, R. microsporus has gained the most attention as the causative agent of rice seedling blight, a plant disease that causes severe crop losses in agriculture in Asia (3). The disease is mediated through the highly potent phytotoxin rhizoxin (Fig. 1), which efficiently stalls plant cell division by binding to the β-tubulin of the rice plant cells. This leads to abnormal swelling of the tips of rice seedling roots, eventually leading to plant death (4). Although R. microsporus was initially believed to be the toxin producer, we discovered that rhizoxin is biosynthesized by an endosymbiotic betaproteobacterium, *Mycetohabitans rhizoxinica* (synonym Burkholderia rhizoxinica), residing within the fungal hyphae (5). *M. rhizoxinica* not only provides the fungus with potent toxins but also regulates fungal reproduction (6, 7). This toxinogenic bacterial-fungal alliance is globally distributed across all five continents inhabiting a variety of niches ranging from temperate and arid soils to human tissue (8, 9). In one of these eight toxin-producing *Rhizopus*-*Mycetohabitans* strains rhizoxin was shown to be a potent phytotoxin (Fig. 1), while the ecological role of rhizoxin in the other *Rhizopus* strains is currently unknown.

Since fungi are able to utilize toxic secondary metabolites to protect themselves from predators and antagonistic organisms (10–12), we reason that rhizoxin might act as an antipredator agent in nonpathogenic *Rhizopus* strains. Effective defense strategies are particularly important for fungi since their high nutrient content, large biomass, and inability to move make fungi an ideal food source for micropredators such as soil-dwelling amoeba, nematodes, mites, and springtails (13–19). For example, the soil mold Aspergillus nidulans relies on secondary metabolites to defend itself against the fungivorous springtail *Folsomia candida* (20, 21), while aflatoxin protects Aspergillus flavus from fungivory by insects (22).

While most studies focus on species belonging to the Ascomycota and Basidiomycota, reports on toxic defense molecules produced by Mucoromycota fungi are scarce. Interestingly, Mucoromycota fungi often harbor endobacteria (23, 24), which can produce toxic secondary metabolites that shield the fungal host from predatory nematodes (25). This strategy to fend off predators might be a common trait in symbiotic Mucoromycota fungi, since all toxic compounds identified in Mucoromycota fungi so far are produced by endofungal bacteria. For example, *M. rhizoxinica* is a fungal endosymbiont with a remarkable potential to produce secondary metabolites (26–29), despite its small genome size (3.75 Mb) (30). Among the many secondary metabolites produced by *M. rhizoxinica*, rhizoxin represents a prime candidate as a potential antipredator agent. Rhizoxin exhibits its effect against most eukaryotes, including vertebrates and fungi by efficiently binding to β-tubulin, which causes disruption of microtubule formation (31, 32).

However, apart from being a potent phytotoxin, the ecological role of rhizoxin is still unclear and raises the question why soilborne fungi harbor toxin-producing bacteria. Here, we tested the effects of rhizoxin on the model nematode Caenorhabditis elegans, and two mycophagous eukaryotes, the amoeba *Protostelium aurantium* and the nematode Aphelenchus avenae. We show that rhizoxin-producing endosymbionts can prevent killing of R. microsporus by protozoan and metazoan fungivorous micropredators.

## RESULTS

### Ingestion of *R. microsporus* spores is toxic to a fungivorous amoeba.

Within the soil community, fungi are constantly challenged by antagonistic organisms such as amoebae, which are well known for their micropredatory lifestyle (33). Using the recently isolated amoeba *Protostelium aurantium*, which specifically feeds on fungi either by phagocytosis of yeast-like cells or by invasion of mature hyphae (15, 34), we investigated whether R. microsporus endosymbionts are able to protect their fungal host from this predatory amoeba.

**FIG 2:**
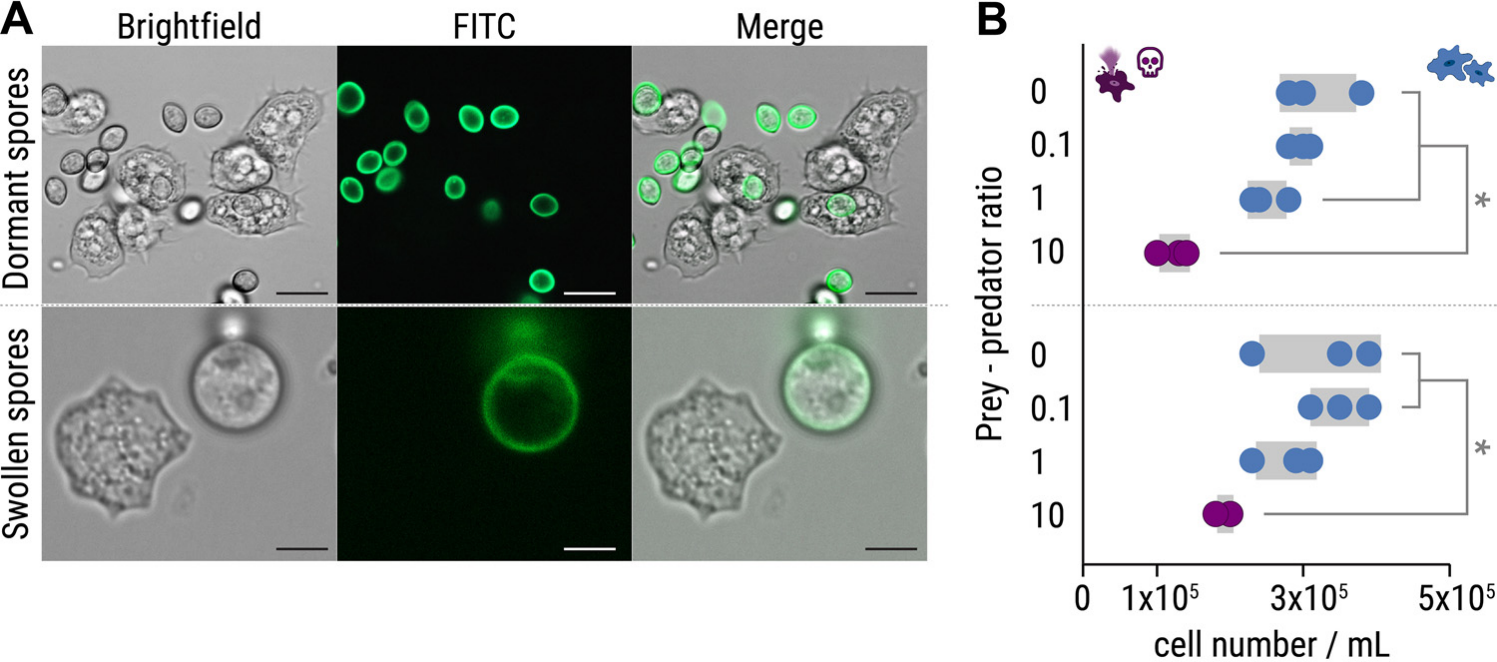
Predation of *Protostelium aurantium* on spores of R. microsporus. (A) Fluorescence microscopy images showing FITC-stained, dormant R. microsporus spores (top) and ingestion of a swollen R. microsporus spore by *P. aurantium* (bottom). Scale bars, 5 μm. (B) Feeding of *P. aurantium* on dormant spores (top) leads to a reduced survival rate of *P. aurantium* compared to swollen spores (bottom). *n* = 3 independent replicated experiments ± 1 SEM. One-way ANOVA was performed with Tukey’s multiple-comparison test (*, *P *< 0.05; see also Table S1B).

In a coculture experiment, *P. aurantium* was first incubated with either dormant or swollen spores of R. microsporus (ATCC 62417). Dormant spores are readily ingested by *P. aurantium*, while swollen spores are taken up less frequently (Fig. 2A; see also Video S1 in the supplemental material [https://doi.org/10.5281/zenodo.6827988]). This difference can be explained by the size of the swollen spores (9.1 μm ± 0.9 μm; see Fig. S1A in the supplemental material), which are significantly larger than dormant spores (5.2 μm ± 0.5 μm, unpaired *t* test: *t *=* *5.93, df = 3.2, *P = *0.0081; see Table S1A). A reduced uptake of swollen R. microsporus spores was previously also reported for macrophages (35). Although the increase in spore size makes it difficult for *P. aurantium* (mean diameter of approximately 13.2 μm ± 2.2 μm) to ingest swollen spores, in some rare cases we observed phagocytosis of swollen spores and R. microsporus germlings (see Fig. S1B). Survival assays revealed that the presence of spores generally reduces the viability of the amoebae at prey predator ratios of 10:1 (*P *< 0.05), with dormant spores being even more harmful for *P. aurantium* than swollen spores (Fig. 2B; see also Table S1B). This sensitivity correlates with a higher frequency of phagocytosis for dormant spores of R. microsporus.

10.1128/mbio.01440-22.2 TABLE S1 (A) Approximate probabilities (*P*) of the unpaired *t* test with Welch’s correction for the mean diameter of dormant and swollen spores (in μm) of R. microsporus ATCC 62417. (B) Approximate probabilities (*P*) of Brown-Forsythe test, one-way ANOVA, and Tukey HSD *post hoc* test for the survival of *Protostelium aurantium* following exposure to dormant and swollen spores of R. microsporus ATCC 62417. Download Table S1, DOCX file, 0.03 MB. Copyright © 2022 Richter et al. 2022 Richter et al. https://creativecommons.org/licenses/by/4.0/ This content is distributed under the terms of the Creative Commons Attribution 4.0 International license.

10.1128/mbio.01440-22.7 FIG S1 (A) Mean diameter of swollen and dormant R. microsporus spores ± 1 SEM (*n* = 59 for dormant spores and *n* = 52 for swollen spores). An unpaired *t* test with Welch’s correction was performed (*, *P *< 0.05; see Table S1A). (B) Predation of *Protostelium aurantium* on germinated spores from R. microsporus. Fluorescence microscopy images showing ingestion of a germinating, swollen R. microsporus spore (stained with FITC) by *P. aurantium*. Scale bars, 5 μm. (C) HPLC profiles of crude extracts from axenic endosymbiotic *Mycetohabitans* showing the two major bacterial rhizoxin congeners (rhizoxin S1 and rhizoxin S2). Samples were monitored at 310 nm. MR, *Mycetohabitans rhizoxinica* HKI-0454; ME, *Mycetohabitans endofungorum* HKI-0456; *ΔrhiG*, rhizoxin-deficient mutant; Ctrl, medium control. Cultures were grown to a similar OD_600_ (~3.5) and extracted. The peak areas were calculated for rhizoxin S1 (*M. rhizoxinica*, 9.07E6; *M. endofungorum*, 5.01E6) and rhizoxin S2 (*M. rhizoxinica*, 2.59E7; *M. endofungorum*, 2.05E7). (D) Amino acid sequence alignment of β-tubulin proteins from amoebae, nematodes, and *Rhizopus* sp. Numbers refer to the amino acid positions from R. microsporus. A black box at amino acid position 100 indicates the positions of the serine or asparagine that confers rhizoxin resistance (black) or sensitivity (white), respectively. Accession numbers for proteins retrieved from the Universal Protein Resource are given in brackets. *, Protein sequences retrieved from GenBank. (E) Microscopic image of A. avenae coincubated with symbiotic R. microsporus showing that the majority of worms are dead (see Video S2 [https://doi.org/10.5281/zenodo.6827988]). Download FIG S1, TIF file, 2.5 MB. Copyright © 2022 Richter et al. 2022 Richter et al. https://creativecommons.org/licenses/by/4.0/ This content is distributed under the terms of the Creative Commons Attribution 4.0 International license.

### Endosymbionts protect fungal host from amoeba predation.

**FIG 3:**
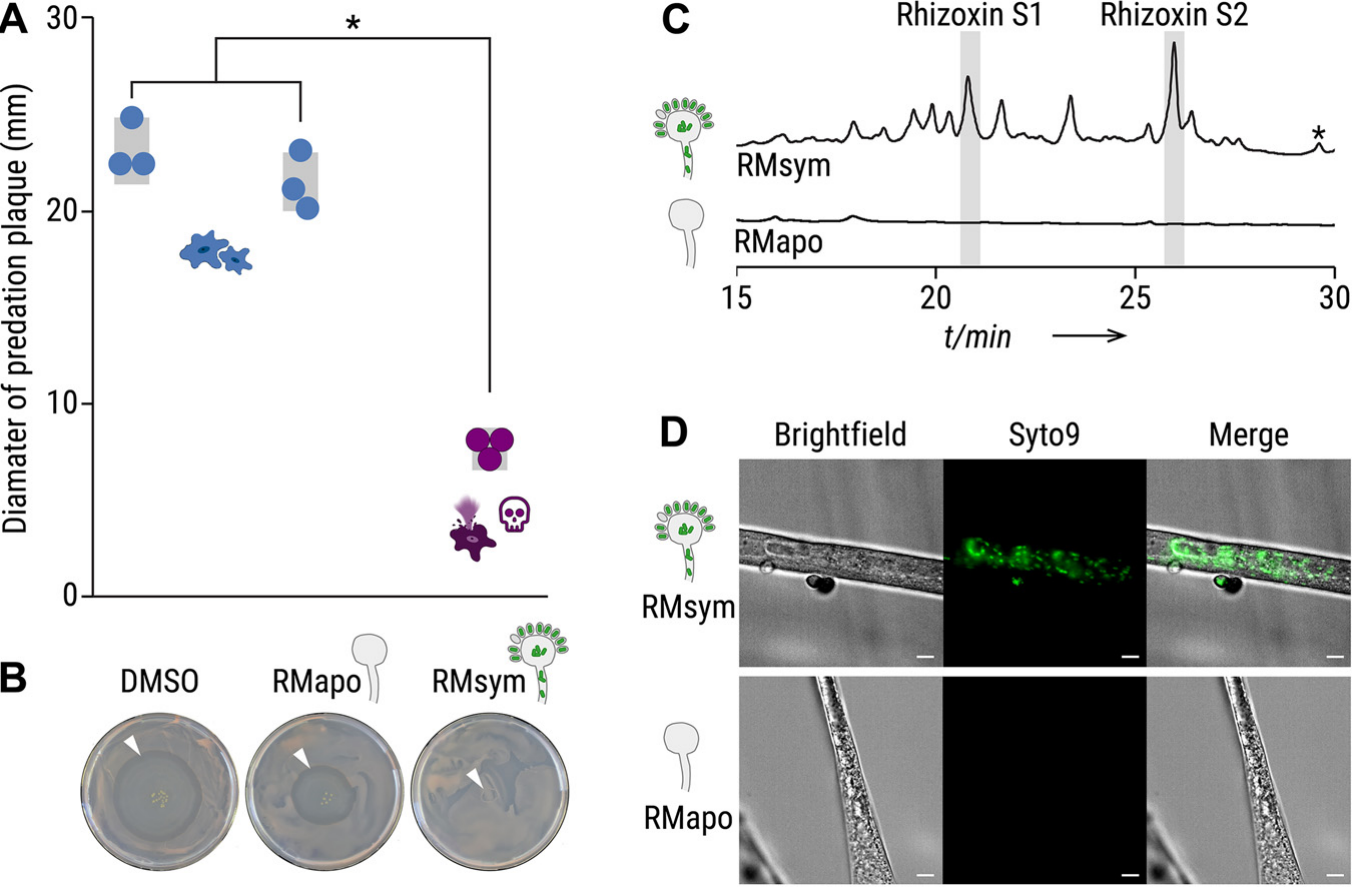
Culture extracts from symbiotic Rhizopus microsporus kills *Protostelium aurantium*. (A) The survival of *P. aurantium*, indicated by the diameter of the predation plaque (clearance of yeast), is significantly reduced in cultures that were exposed to 2% crude culture extract from symbiotic R. microsporus (RMsym). Incubation with solvent alone (DMSO) or apo-symbiotic R. microsporus (RMapo) has no effect on the viability of *P. aurantium.* Circles indicate independent replicated experiments (*n* = 3) ± 1 SEM (gray bars). One-way ANOVA with Tukey’s multiple-comparison test was performed (*, *P *< 0.0001; see Table S2). (B) Photographs of yeast agar plates showing the predation plaque by *P. aurantium* (arrowheads). (C) HPLC profiles of crude extracts from symbiotic and endosymbiont-free R. microsporus showing a mixture of rhizoxin derivatives, including the two major bacterial rhizoxin congeners (rhizoxin S1 and rhizoxin S2). The peak correlating to rhizoxin is marked with an asterisk (*). Monitoring was done at 310 nm (see Fig. S2). The peak areas were integrated to calculate the concentration of rhizoxin S1 (1.2 μM) and rhizoxin S2 (1.7 μM), as well as all rhizoxin congeners combined (8.7 μM). (D) Fluorescence microscopy images of symbiotic R. microsporus and endosymbiont-free R. microsporus. Green fluorescence indicates presence of endosymbionts (SYTO9). Scale bars, 5 μm.

To test whether bacterial endosymbionts are responsible for amoeba killing, trophozoites of *P. aurantium* were exposed to the following fungal culture extracts: (i) symbiotic (endosymbiont-containing) R. microsporus ATCC 62417 (RMsym) and (ii) aposymbiotic R. microsporus ATCC 62417/S (RMapo). Amoebae were coincubated with 2% of the culture extracts for 1 h. Following exposure, the treated samples were placed on agar plates containing Rhodotorula mucilaginosa as a food source. Living amoebae, incubated with solvent control, form a visible predation plaque (clearance of yeast) that expands over 5 days of incubation (Fig. 3A and B). Increasing predation plaques also appeared when amoebae were incubated with extracts from aposymbiotic R. microsporus hyphae, but predation plaques were significantly lower when incubated with crude extracts from symbiotic R. microsporus (*P *< 0.0001, Fig. 3A and B; see also Table S2). This confirms that *P. aurantium* is inhibited or killed due to the presence of endosymbionts.

10.1128/mbio.01440-22.3 TABLE S2 Approximate probabilities (*P*) of the Brown-Forsythe test, one-way ANOVA, and Tukey HSD *post hoc* test for the survival of *Protostelium aurantium* following exposure to 2% crude culture extract from symbiotic R. microsporus (RMsym), endosymbiont-free R. microsporus (RMapo), or solvent control (DMSO). Homogeneous data (nonsignificant Brown-Forsythe) are shown as black numbers, and nonhomogeneous data (significant Brown-Forsythe) are highlighted as red numbers. *P* values of <0.05 were considered statistically significant (highlighted in grey). Download Table S2, DOCX file, 0.03 MB. Copyright © 2022 Richter et al. 2022 Richter et al. https://creativecommons.org/licenses/by/4.0/ This content is distributed under the terms of the Creative Commons Attribution 4.0 International license.

10.1128/mbio.01440-22.8 FIG S2 Characterization of rhizoxin derivatives. The identity of rhizoxin S1 and rhizoxin S2 in culture extracts from R. microsporus (A) and *M. rhizoxinica* (B) was verified by comparison to authentic references (C) using high-resolution mass spectrometry and UV spectra. Download FIG S2, TIF file, 1.9 MB. Copyright © 2022 Richter et al. 2022 Richter et al. https://creativecommons.org/licenses/by/4.0/ This content is distributed under the terms of the Creative Commons Attribution 4.0 International license.

### The amoebicidal effect is delivered by rhizoxin.

Since *M. rhizoxinica* has a remarkable potential to produce toxic secondary metabolites, we investigated whether killing of the amoeba is caused by a bacterial metabolite. The fungal culture extracts were analyzed for the presence of derivatives of the known cytotoxic macrolide rhizoxin (5). HPLC profiles revealed a mixture of rhizoxin derivatives, including the two major bacterial rhizoxin congeners (rhizoxin S1 and rhizoxin S2), in the symbiotic R. microsporus extract (Fig. 3C; see also Fig. S2) (36). The presence of *M. rhizoxinica* in the mycelium of symbiotic R. microsporus was confirmed by fluorescence microscopy (Fig. 3D). In comparison, bacterial cells were absent in endosymbiont-free R. microsporus mycelium, and none of the rhizoxin congeners were detected, suggesting that bacterial rhizoxins may cause lethal effects when ingested by *P. aurantium.*

**FIG 4:**
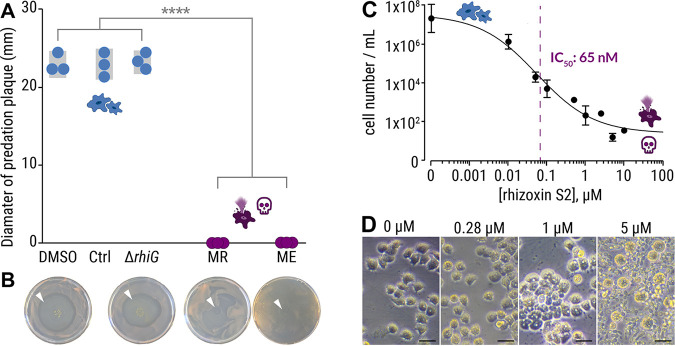
Culture extracts from axenic *Mycetohabitans* sp. kill *Protostelium aurantium*. (A) The viability of *P. aurantium*, indicated by the diameter of the predation plaque (clearance of yeast), is significantly reduced in cultures that were exposed to 2% crude culture extract from axenically grown endosymbiotic *M. rhizoxinica* HKI-0454 (labeled MR) or *M. endofungorum* HKI-0456 (labeled ME). Incubation with solvent alone (DMSO), extract from culture medium (Ctrl), or rhizoxin-deficient *M. rhizoxinica* (Δ*rhiG*) has no effect on the viability of *P. aurantium.* Circles indicate independent replicated experiments (*n* = 3) ± 1 SEM (gray bars). One-way ANOVA with Tukey’s multiple-comparison test was performed (****, *P *< 0.0001; see Table S3). (B) Photographs of yeast agar plates showing the predation plaque by *P. aurantium* (arrowheads). (C) Liquid survival assay of *P. aurantium* supplemented with the bacterial rhizoxin S2. Data points represent three independent replicated experiments (*n* = 3) ± 1 SEM. (D) Microscopic images showing the growth of *P. aurantium* in the presence of rhizoxin S2. Scale bars, 20 μm.

To clarify whether the amoebicidal effect is delivered by a bacterial metabolite, we subjected *P. aurantium* to crude culture extracts from two representative endosymbiotic *Mycetohabitans* species, *M. rhizoxinica* (Fig. 4A, MR) and *M. endofungorum* (Fig. 4A, ME), which were isolated from their respective R. microsporus hosts (R. microsporus ATCC 62417 and R. microsporus CBS112285, Fig. 1) (8, 37). Both culture extracts from axenically grown bacteria kill all trophozoites of *P. aurantium* (Fig. 4A and B). This effect is significant compared to culture extracts from a rhizoxin-deficient *M. rhizoxinica* strain (*ΔrhiG*) and solvent controls (*P < *0.0001, Fig. 4A and B; see also Table S3). HPLC analysis confirmed the presence of rhizoxin congeners in the two bacterial extracts, whereas none of the congeners are detected in extracts from the rhizoxin-deficient *M. rhizoxinica* strain (see Fig. S1C).

10.1128/mbio.01440-22.4 TABLE S3 Approximate probabilities (*P*) of the Brown-Forsythe test, one-way ANOVA, and Tukey HSD *post hoc* test for the survival of *Protostelium aurantium* following exposure to 2% crude extract from axenic endosymbiotic *Mycetohabitans rhizoxinica* HKI-0454 (Mr), *Mycetohabitans endofungorum* HKI-0456 (Me), rhizoxin-deficient mutant (*ΔrhiG*) cultures, solvent control (DMSO), and untreated control. Homogeneous data (nonsignificant Brown-Forsythe) are shown as black numbers, and nonhomogeneous data (significant Brown-Forsythe) are is highlighted as red numbers. *P* values of <0.0001 were considered statistically significant (highlighted in gray). Download Table S3, DOCX file, 0.03 MB. Copyright © 2022 Richter et al. 2022 Richter et al. https://creativecommons.org/licenses/by/4.0/ This content is distributed under the terms of the Creative Commons Attribution 4.0 International license.

To test whether bacterial rhizoxins kill *P. aurantium*, one of the major bacterial rhizoxin congeners (rhizoxin S2) was isolated from *M. rhizoxinica* as described previously (36) and its potency was assessed in a liquid *P. aurantium* survival assay (50% inhibitory concentration [IC_50_] = 58 nM, 95% confidence interval [CI] = 35 to 100 nM; Fig. 4C). The calculated IC_50_ value is 2 orders of magnitude lower than the estimated concentration of rhizoxin S2 in both bacterial culture extracts (*M. rhizoxinica*, 4.7 μM; *M. endofungorum*, 1.7 μM; see Fig. S1C), which is in line with the observation that not a single *P. aurantium* cell survived the treatment with bacterial culture extracts (Fig. 4A). The sensitivity of *P. aurantium* is comparable to the cytotoxic concentration against human HeLa cells previously reported for rhizoxin S2 (50% cytotoxic dose = 239 nM) (36). These results were confirmed by microscopic images, showing a slight change in morphology of amoeba exposed to 280 nM rhizoxin S2. At this concentration, amoeba cells start to aggregate with some cells changing their appearance to a rounded shape. These typical signs of starvation become more prevalent with increasing concentrations of rhizoxin S2 (1 μM). At 5 μM rhizoxin S2, all amoeba cells are dead (completely round), and the food source (yeast cells) remains unconsumed (Fig. 4D). These results confirm that bacterial rhizoxins are responsible for the killing of the fungivorous amoeba *P. aurantium*.

### Endosymbionts protect *R. microsporus* from soil-dwelling nematodes.

In addition to amoeba, fungi are also challenged by metazoan micropredators within the soil community. Since nematodes are among the most abundant metazoan of the soil community (38), we investigated whether bacterial endosymbionts can protect R. microsporus from the ubiquitous soil nematode C. elegans, which has become a model system for studying host-pathogen interactions (39).

**FIG 5:**
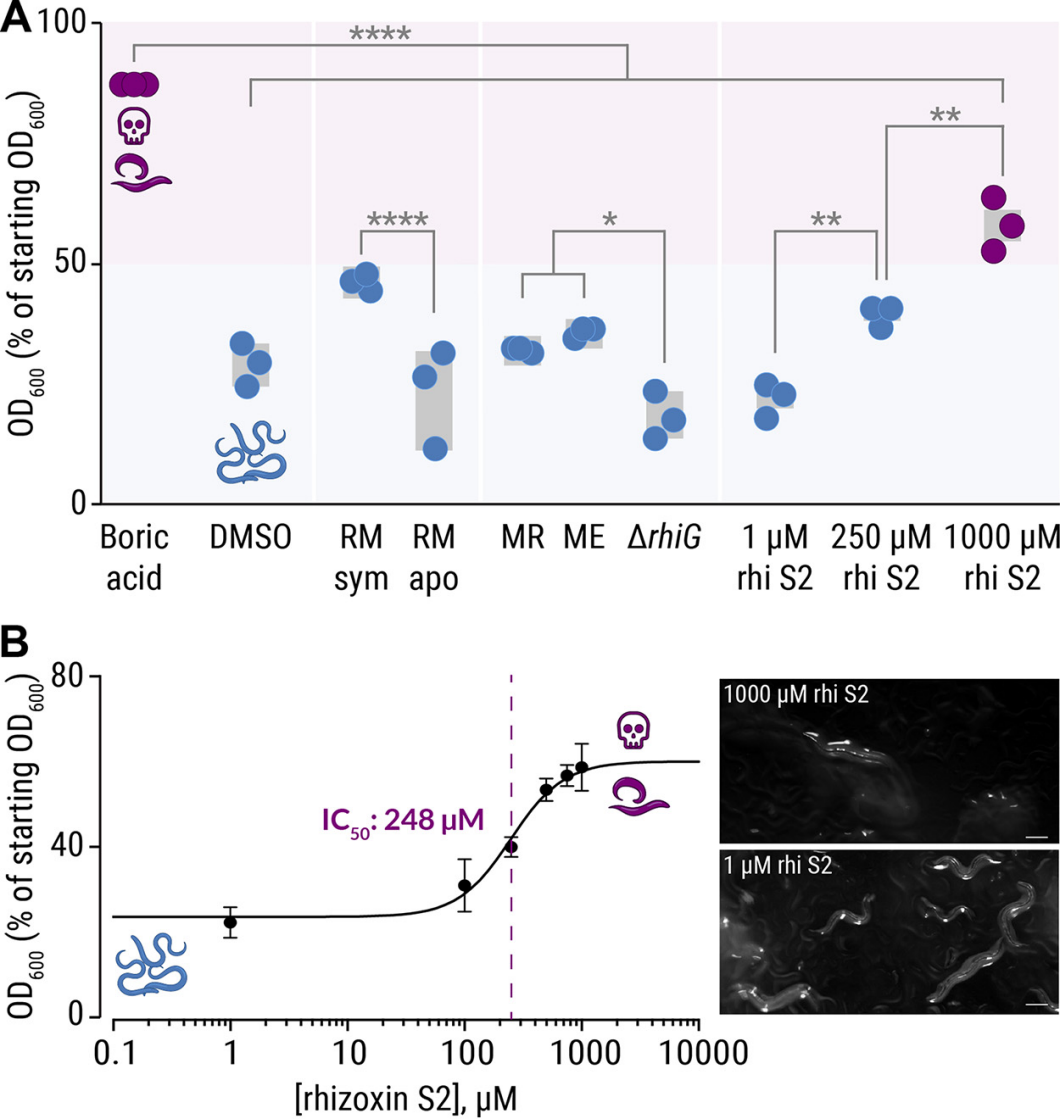
Inhibitory effects of crude extracts and pure rhizoxin S2 on C. elegans. (A) C. elegans, coincubated with E. coli OP50 cells as food source, were exposed to 2% crude culture extracts from symbiotic R. microsporus (RMsym), endosymbiont-free Rhizopus microsporus (RMapo), axenically grown endosymbiotic *M. rhizoxinica* HKI-0454 (labeled MR), *Mycetohabitans endofungorum* HKI-0456 (labeled ME), and rhizoxin-deficient *M. rhizoxinica* (Δ*rhiG*), as well as pure rhizoxin S2 (rhi S2). Since the number of viable nematode worms in the suspension is directly related to the E. coli cell density, the OD_600_ values were plotted as a percentage of the starting OD_600_. Incubation with 18 mM boric acid (positive control) kills most of the nematodes (E. coli density of 80%), while exposure to crude culture extracts has a mild effect on C. elegans viability. Circles indicate independent replicated experiments (*n* = 3) ± 1 SEM (gray bars). One-way ANOVA with Tukey’s multiple-comparison test was performed (*, *P *< 0.03; **, *P* < 0.002; ****, *P* < 0.0001; see Table S4). (B) Liquid feeding inhibition assay of C. elegans supplemented with the bacterial rhizoxin S2. Data points represent three independent replicated experiments (*n* = 3) ± 1 SEM. Microscopic images of nematodes exposed to pure rhizoxin S2 are shown. Scale bars, 200 μm.

C. elegans, cocultured with E. coli OP50 as a food source, was exposed to a 2% culture extract in a liquid feeding inhibition assay. The number of viable nematode worms in the suspension is directly related to the E. coli cell optical density at 600 nm (OD_600_). The positive control, 18 mM boric acid, kills the majority of worms (indicated by high OD_600_ values). As expected, the solvent control (dimethyl sulfoxide [DMSO]) has no effect on nematode viability (indicated by low OD_600_ values). Although the culture extracts are not as potent as the positive control, significant differences in nematode viability are observed between various extracts. For example, markedly fewer nematodes survive treatment with the symbiotic R. microsporus extract (~50% of nematodes are dead) compared to the extract from endosymbiont-free R. microsporus (*P < *0.0001; Fig. 5A; see also Table S4), which is consistent with the effect of the fungal extracts on the survival of *P. aurantium* (Fig. 2A). Surprisingly, bacterial extracts (*M. rhizoxinica* and *M. endofungorum* strains) show a minor effect on the survival rate of C. elegans. However, significantly more worms die when treated with either *M. rhizoxinica* or *M. endofungorum* extracts compared to an extract from bacteria that are unable to produce rhizoxin (*ΔrhiG*) (*P *< 0.03; Fig. 5A; see also Table S4).

10.1128/mbio.01440-22.5 TABLE S4 Approximate probabilities (*P*) of the Brown-Forsythe test, one-way analysis of variance (ANOVA), and Tukey HSD *post hoc* test for the survival of C. elegans following exposure to 2% crude culture extract from symbiotic R. microsporus (RMsym), endosymbiont-free R. microsporus (RMapo), axenic endosymbiotic *Mycetohabitans rhizoxinica* HKI-0454 (labeled MR), *Mycetohabitans endofungorum* HKI-0456 (labeled ME), rhizoxin-deficient mutant (*ΔrhiG*) cultures, and various concentrations of pure rhizoxin S2 (rhi S2). The following controls were included in each experiment: extract of culture medium (Control), solvent control (DMSO), reactions not containing C. elegans (-N2WT), and boric acid as a positive control. Homogeneous data (nonsignificant Brown-Forsythe) are shown as black numbers, and nonhomogeneous data (significant Brown-Forsythe) are highlighted as red numbers. *P* values of <0.05 were considered statistically significant (highlighted in gray). Download Table S4, DOCX file, 0.04 MB. Copyright © 2022 Richter et al. 2022 Richter et al. https://creativecommons.org/licenses/by/4.0/ This content is distributed under the terms of the Creative Commons Attribution 4.0 International license.

Since it was previously shown that the mobility of C. elegans decreases when directly in contact with *M. rhizoxinica* (40), we tested various concentrations of pure rhizoxin S2 (1 to 1,000 μM) in a liquid C. elegans feeding inhibition assay. The viability of nematodes significantly decreases as the concentration of rhizoxin S2 increases (*P *< 0.002, Fig. 5A; see also Table S4), with a half-maximal inhibitory concentration of 248 μM (95% CI = 187 to 329 μM; Fig. 5B). The effect of 250 μM pure rhizoxin S2 on nematode survival is comparable to the effects of crude culture extracts (Fig. 5A) suggesting that other natural compounds are produced that have an inhibitory or toxic effect on C. elegans. The calculated IC_50_ value is 2 orders of magnitude higher than the estimated concentration of rhizoxin S2 in both bacterial culture extracts (*M. rhizoxinica*, 4.7 μM; *M. endofungorum*, 1.7 μM; see Fig. S1C), which is in line with the observation that the bacterial crude culture extracts have a minor effect on the survival rate of C. elegans (Fig. 5A). The inhibitory concentration of rhizoxin S2 against C. elegans (248 μM), exceeds the one found for *P. aurantium* (IC_50_ = 58 nM) by 4 orders of magnitude. The comparably low toxicity of pure rhizoxin S2 toward this nematode may be explained by the mode of delivery as rhizoxin is not directly ingested by C. elegans. Instead, toxin delivery depends on diffusion through a tough exterior cuticle, which may impose a barrier to efficient rhizoxin uptake (41).

Since the bacterivorous C. elegans does not feed on fungi in its natural environment, we investigated the interaction between the fungivorous nematode A. avenae and R. microsporus. *A. avenae* was chosen as a model micropredator because of its ability to feed on fungal hyphae (42). In addition, *A. avenae* and R. microsporus share the same ecological niche, since both species are globally distributed and inhabit temperate soils (8, 42).

**FIG 6:**
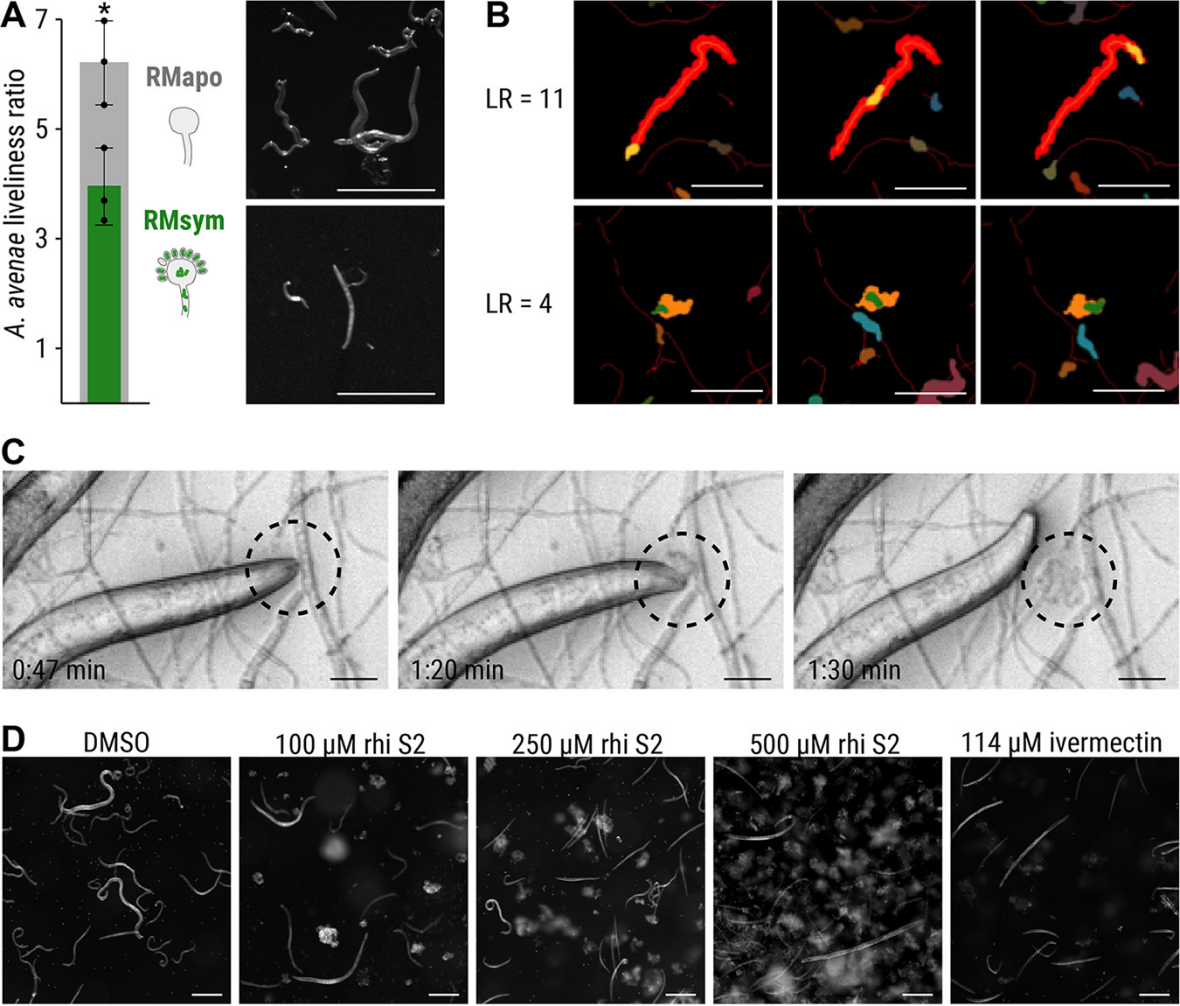
Feeding inhibition of A. avenae on R. microsporus. (A) *A. avenae* was coincubated with symbiotic R. microsporus (RMsym) or endosymbiont-free R. microsporus (RMapo) for 2 to 3 weeks. Nematode movement was recorded using a stereomicroscope with a frame rate of 1 fps. The liveliness of the worms was calculated from the ratio of the area covered by a worm, divided by the area of the worm itself, and scaled to the full length of the movie. The minimum scaled liveliness ratio (LR) for a live worm was set to 1.5, below this value the worm was declared inactive/dead. *n* = 3 independent replicated experiments ± 1 SEM. An unpaired *t* test with Welch’s correction was performed (*, *P *< 0.05; see Table S5). Microscope images of *A. avenae* used for analysis. Scale bars, 500 μm. (B) Illustrations of the LR at high (top) and medium (bottom) values. (Top) The worm shown in orange covers the red footprint area during the time course of the experiment. These images show the first (left column), middle (middle column), and final (right column) time points of the movie. The activity of a worm was characterized by dividing the endpoint footprint by the area of the worm at each time point. The resulting LR was 11.5 for the worm in the top row, thus indicating a very active nematode. (Bottom) A less active worm (green area) covered a smaller footprint (orange area), as shown by the LR value of 4.0. Scale bars, 300 μm. See the live videos of the segmented worms and their footprints in Videos S11 and S12 (https://doi.org/10.5281/zenodo.6827988) for the worms with LR = 11.5 and LR = 4.0, respectively. (C) Time-lapse images of *A. avenae* feeding on endosymbiont-free R. microsporus (black circle). Endosymbiont-free R. microsporus ATCC 62417/S was coincubated with *A. avenae* for 24 h in a microchannel slide (Ibidi), and feeding was recorded on a spinning disc microscope (see Movie S1). Scale bars, 20 μm. No feeding was observed in worms that were coincubated with symbiotic R. microsporus (see Fig. S1D; see also Video S2 [https://doi.org/10.5281/zenodo.6827988]). (D) Microscopic images of *A. avenae* after exposure to different concentrations of pure rhizoxin S2 (rhi S2). Worms were healthy and alive when exposed to the solvent control (DMSO). Exposure to 114 μM ivermectin killed all worms. See the live videos of nematode movement in Videos S3 to S7 (https://doi.org/10.5281/zenodo.6827988). Scale bars, 200 μm.

To study the feeding behavior of *A. avenae*, nematodes were cultured on symbiotic R. microsporus as well as endosymbiont-free R. microsporus for 3 to 4 weeks. After coincubation, nematodes were harvested, and their viability was assessed by calculating their liveliness ratio (LR) as a measure of fitness. The LR was defined as the ratio of the area covered by a worm, divided by the area of the worm itself, and scaled to the full length of the time-lapse movie (30 s). Nematodes with LR values below 1.4 were considered immobile, whereas a fast-moving worm would be characterized by a high LR value (the faster the worm’s movement, the higher the LR value). Nematodes grown on aposymbiotic R. microsporus are healthy and active, as indicated by an LR of 6.24 ± 0.64, while worms living on symbiotic R. microsporus are significantly less lively (LR = 3.96 ± 0.57, *P *< 0.05; Fig. 6A and B; see also Table S5). Coincubation of *A. avenae* with aposymbiotic R. microsporus in a microchannel slide confirmed active feeding of *A. avenae* on R. microsporus lacking endosymbionts (Fig. 6C; see also Movie S1). *A. avenae* pierces the fungal cell wall with a stylet and feeds on the fungal cytoplasm by sucking (43). Sucking is facilitated by muscle contractions of the esophagus, which is clearly visible in Movie S1. After feeding, the nematode leaves a wound in the fungal cell wall, which leads to the release of fungal cytoplasm into the microchannel (Fig. 6C; see also Movie S1). In contrast, we did not observe active feeding on symbiotic R. microsporus hyphae, with the majority of the worms being dead (see Fig. S1D and Video S2 [https://doi.org/10.5281/zenodo.6827988]). To investigate whether the protective effect against *A. avenae* is mediated through rhizoxin, *A. avenae* was exposed to various concentrations of rhizoxin S2 (0 to 500 μM) in a liquid toxicity assay. Microscopic analysis shows a reduced number of worms for all three rhizoxin S2 concentrations compared to the solvent control (Fig. 6D). Worms are alive and healthy following exposure to 100 μM rhizoxin S2, although the number of worms is reduced compared to the solvent control. Exposure to 250 μM rhizoxin S2 resulted in the majority of worms being dead, while treatment with 500 μM rhizoxin S2 kills all worms similar to the positive control (114 μM ivermectin; see Videos S3 to S7 [https://doi.org/10.5281/zenodo.6827988]). Combined with our C. elegans data (IC_50_ = 248 μM), these nematicidal concentrations of rhizoxin S2 are similar to the growth inhibiting concentration of the plant-pathogenic fungus *Phytophthora ramorum* previously reported for various rhizoxin congeners (1.6 μM) (44).

10.1128/mbio.01440-22.1 MOVIE S1 A. avenae feeding on endosymbiont-free R. microsporus. Endosymbiont-free R. microsporus ATCC 62417/S was coincubated with *A. avenae* for 24 h in a microchannel slide (Ibidi), and feeding was recorded on a spinning disc microscope. Download Movie S1, AVI file, 5.0 MB. Copyright © 2022 Richter et al. 2022 Richter et al. https://creativecommons.org/licenses/by/4.0/ This content is distributed under the terms of the Creative Commons Attribution 4.0 International license.

10.1128/mbio.01440-22.6 TABLE S5 Approximate probabilities (*P*) of an unpaired *t* test with Welch’s correction for the survival (liveliness ratio) of A. avenae grazing on endosymbiont-free R. microsporus (column A) or symbiotic R. microsporus (column B). Download Table S5, DOCX file, 0.03 MB. Copyright © 2022 Richter et al. 2022 Richter et al. https://creativecommons.org/licenses/by/4.0/ This content is distributed under the terms of the Creative Commons Attribution 4.0 International license.

**FIG 7:**
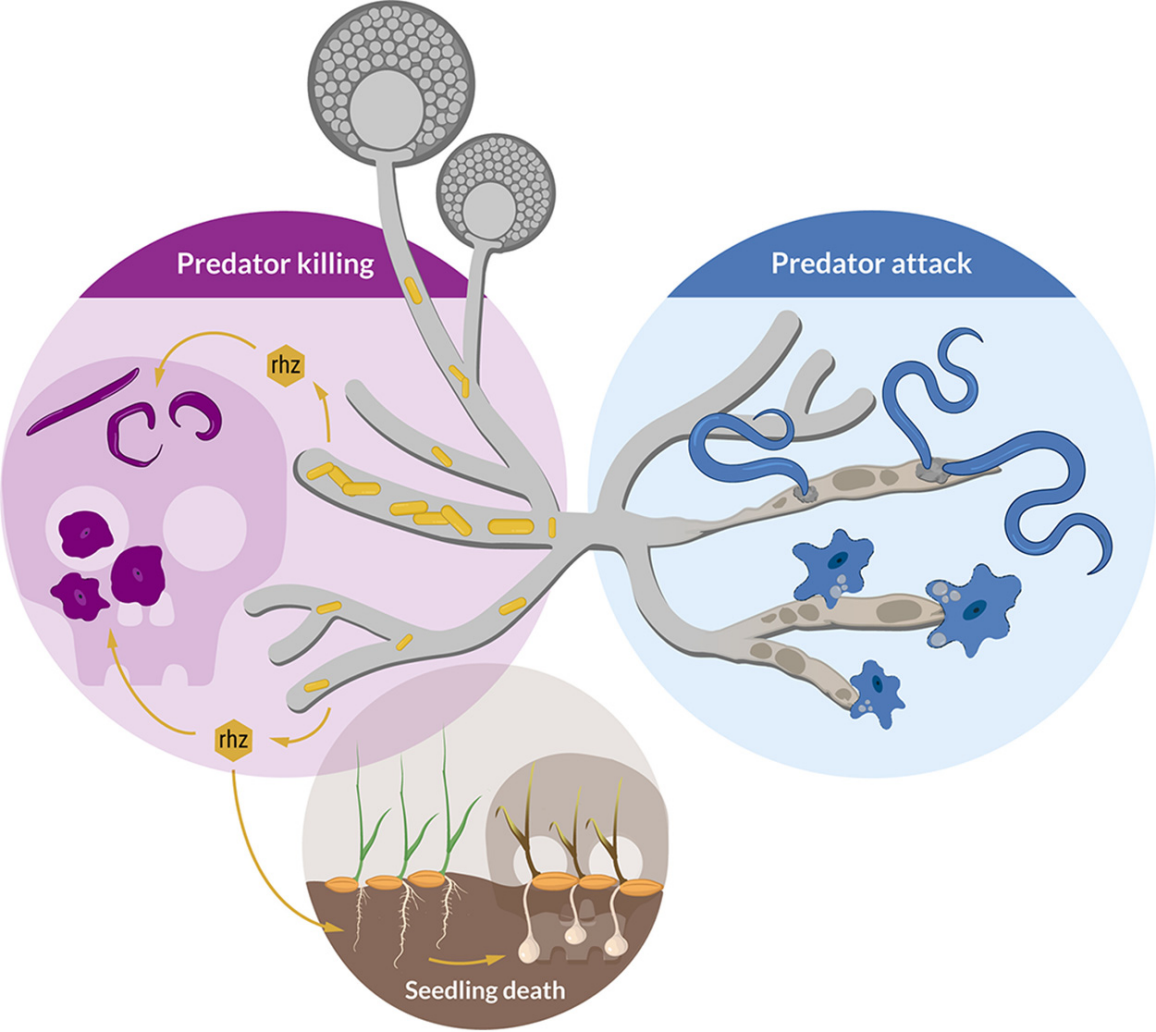
Schematic model of the ecological role of rhizoxin-producing endofungal bacteria (*M. rhizoxinica*). The fungal host (Rhizopus microsporus) utilizes the bacterial secondary metabolite rhizoxin to fend off fungivorous micropredators such as amoeba and nematodes. The absence of endofungal bacteria leads to R. microsporus being attacked and subsequently killed by protozoan and metazoan predators. The establishment of the *Rhizopus*-*Mycetohabitans* symbiosis may have first developed to provide protection against fungal predators, with the emergence of plant pathogenicity developing later.

These results confirm that R. microsporus is protected from *A. avenae* through the secondary metabolite rhizoxin S2, which is produced by endosymbionts living within the fungal hyphae (Fig. 7). In line with this model, *A. avenae* is likely rhizoxin sensitive, since the β-tubulin amino acid sequence of the closely related fungivorous nematode *Bursaphelenchus okinawaensis* harbors asparagine at amino acid position 100 (see Fig. S1E). These results highlight a defensive function of an endofungal symbiotic bacterium against protozoan and metazoan predators.

## DISCUSSION

Prey-predator interaction is a major driver of biodiversity (45). Within the soil microbiome, fungi are constantly threatened by fungivorous organisms selecting for strategies to defend themselves against predators (10, 11, 46). In this study, we revealed that endosymbionts protect the phytopathogenic fungus R. microsporus against fungivorous protozoan and metazoan predators. Using a combination of coculture experiments, survival assays, and fluorescence microscopy, we report an amoebicidal and nematicidal effect of rhizoxin, a secondary metabolite produced by the bacterium *M. rhizoxinica* residing within the hyphae of R. microsporus (47).

Fungivorous amoebae are ubiquitous in soil and leaf litter with *P. aurantium* being a prime example of a species that feeds on a wide range of unicellular yeasts, as well as conidia and hyphae of filamentous fungi (15, 34). We observed that *P. aurantium* also ingests spores of R. microsporus via phagocytosis, as shown by fluorescence microscopy. However, ingestion of spores has lethal consequences for the amoeba when R. microsporus spores contain the toxin-producing endosymbiont (5). Although it was previously suggested that Ralstonia pickettii, an endosymbiont of R. microsporus, secretes growth suppressing factors against the soil-dwelling amoeba Dictyostelium discoideum (35), we present the first report of rhizoxin-mediated killing of a fungivorous amoeba. These results highlight that endosymbionts protect their fungal host from being attacked by mycophagous amoeba. Rhizoxin toxicity is most likely not limited to *P. aurantium*, but it may have a rather wide biological range among the kingdom of Amoebozoa, since similar effects have been observed for the distantly related parasitic amoeba Entamoeba histolytica, whose survival rate was reduced by 58% when exposed to methanol extracts (1 g/L) from R. microsporus cultures (48).

Rhizoxin efficiently binds to β-tubulin, leading to the potent depolarization of microtubules and subsequent mitotic arrest in humans and plants (49). A conserved residue at amino acid position 100 of the β-tubulin protein is important for rhizoxin binding and subsequently rhizoxin sensitivity (32). In line with these observations, the *P. aurantium* β-tubulin protein harbors the amino acid asparagine at position 100 (see Fig. S1E), which is an important feature in rhizoxin-sensitive fungi (32). Depolarization of microtubule in amoeba has severe consequences, as β-tubulins have been shown to be essential parts of the microtubule network in D. discoideum, important for cell polarity, migration, and the movement of intracellular particles (50). Intriguingly, *P. aurantium* requires substantial condensation of actin filaments when grabbing and invading the rather large fungal hyphae (15). It is thus conceivable that this part of the cytoskeleton, which actually enters fungal cells would be a primary and highly effective target of the intrafungal rhizoxin.

We further found that rhizoxin can also protect against higher eukaryotic predators from the kingdom of Metazoa. Feeding inhibition assays revealed a lethal effect of this compound on the soil-dwelling model nematode C. elegans and the fungivorous nematode *A. avenae.* The inhibitory concentration is far higher than that for *P. aurantium*. This relatively low sensitivity toward rhizoxin S2 may be due to the sophisticated xenobiotic metabolism and transport systems of C. elegans, which can alter the availability of certain compounds (51). In addition, rhizoxin might lose its activity when ingested by nematodes since acidic conditions, which are present in the posterior intestine of C. elegans (pH of 3.6), cause degradation of the molecule (36, 52). However, the results presented here are in line with previous reports showing that the potencies of different rhizoxin congeners can vary greatly between organisms. For example, picomolar concentrations of rhizoxin S2 inhibit proliferation of leukemia cell lines (50% growth inhibition 1.6 pM), while growth inhibition of diverse fungal strains is caused by rhizoxin concentrations in the micromolar to millimolar range (32, 36, 44). Thus, the effectiveness against both nematode species fits within the range of potencies reported for rhizoxin and may greatly depend on the biological setting. While *P. aurantium* and *A. avenae* actively feed on the fungal cytoplasm, C. elegans is bacterivorous and thus unlikely to be directly exposed to toxins from fungal endosymbionts. However, the alignment of all six C. elegans β-tubulin amino acid sequences suggests that also C. elegans is sensitive to rhizoxin due to the presence of asparagine at amino acid position 100 (see Fig. S1E). In contrast to the susceptible tubulins of the micropredators, the R. microsporus β-tubulin amino acid sequence contains serine at amino acid position 100 which conveys rhizoxin resistance to the fungal host (32). Since worm tubulins play an essential role during all phases of the cell and life cycle (53), it is likely that rhizoxin-induced killing of C. elegans is mediated through microtubule depolarization.

The combination of stereomicroscopy, automated image analysis, and mathematical quantification of nematode movement demonstrated that the fungivorous nematode *A. avenae* actively feeds on R. microsporus that is lacking endosymbionts. In contrast, presence of the bacterial endosymbionts in symbiotic R. microsporus causes death in the majority of worms. This lethal effect is either due to nematode starvation or active feeding on R. microsporus and subsequent ingestion of toxic compounds. However, since C. elegans does not feed on axenic *M. rhizoxinica* leading to nematode death by starvation (40), it is likely that the presence of *Mycetohabitans* spp. inside the fungal hyphae protects R. microsporus from predatory nematodes.

These results highlight a defensive function of an endofungal symbiotic bacterium against a metazoan predator and may explain the very limited *A. avenae* range of prey among Mucoromycota fungi with only two species (*Mucor hiemalis* and *Mortierella verticillata* strain CBS 225.35) known to act as a food source (25, 54). Interestingly, these strains contain non-toxin-producing endosymbionts (25, 55). In contrast, bacterial endosymbionts of the fungal strain *M. verticillata* NRRL 6337 produce toxic metabolites that protect the fungal host from fungivorous nematodes (25). Since Mucoromycota fungi often contain endobacteria (56), host protection through endobacterial metabolites may be more common than previously thought.

By discovering fungivorous predation on R. microsporus this study adds another dimension to the tripartite interaction between fungal host, symbiont, and rice plants (2) and opens an interesting evolutionary perspective. The establishment of the *Rhizopus*-*Mycetohabitans* symbiosis may have originally developed to provide protection against fungal predators and only later facilitated the emergence of plant pathogenicity. The interactions between Mucoromycota and their bacterial endosymbionts are an ancient phenomenon dating back as far as 400 million years (57). Flowering land plants such as rice developed far later (134 million years ago) than protozoan and metazoan predators such as amoebae and nematodes (400 million years ago) (58, 59). Thus, the establishment of a mutualistic interaction between *Rhizopus* and rhizoxin-producing *Mycetohabitans* (60) may have allowed the fungal host to evade predator attack, thereby gaining an evolutionary advantage over aposymbiotic or rhizoxin-negative symbiotic fungi.

Here, we revealed an unexpected role for rhizoxin. In addition to causing blight disease in rice seedlings, we show that this bacterial secondary metabolite is utilized by the fungal host to successfully fend off micropredators (Fig. 7). This antipredator effect of toxin-producing endofungal bacteria of *Rhizopus* is an important addition to a similar observation in *Mortierella* species (25) and points to a more widespread ecological role of endosymbionts. In line with this model, endosymbiont-containing *Rhizopus* species are globally distributed, and the toxin produced by the endosymbionts is lethal to most eukaryotes, including insects, vertebrates, and fungi. It is thus conceivable that animal predation represents an evolutionary driving force to maintain endosymbionts in nonpathogenic fungi.

## MATERIALS AND METHODS

### Strains and growth conditions.

Liquid cultures of *P. aurantium* var. *fungivorum* were grown to confluence in 2 mM phosphate buffer (PB; pH 6.2) supplemented with Rhodotorula mucilaginosa as a food source in standard-sized petri dishes at 22°C (34).

The endobacteria *M. rhizoxinica* HKI-0454 and *M. endofungorum* HKI-0456 were isolated from the mycelia of Rhizopus microsporus ATCC 62417 and Rhizopus microsporus CBS112285, respectively (8, 37). Axenic *Mycetohabitans* cultures were maintained at 30°C in MGY M9 medium (10 g/L glycerol, 1.25 g/L yeast extract, M9 salts) or standard I nutrient agar (Merck, Darmstadt, Germany) supplemented with 1% glycerol. Symbiotic R. microsporus ATCC 62417 (RMsym) was treated with antibiotics to eliminate its endosymbionts (61), resulting in the aposymbiotic fungal strain ATCC 62417/S (RMapo). Since the lack of endosymbionts also abolishes the production of rhizoxin, we confirmed the absence of symbionts from ATCC 62417/S by checking for rhizoxin congeners in ATCC 62417/S culture extracts via high-pressure liquid chromatography (HPLC) (36). Both R. microsporus strains (ATCC 62417 and ATCC 62417/S) were cultivated on potato dextrose agar (PDA; Becton, Dickinson, & Company, Sparks, MD) at 30°C.

C. elegans wild-type N2 (var. Bristol), purchased from the C. elegans Genetics Centre (CGC; University of Minnesota), was grown and maintained on nematode growth medium (NGM) containing E. coli OP50 as a food source (62).

### Fluorescence microscopy of *R. microsporus*.

One-week-old fungal cultures (symbiotic R. microsporus ATCC 62417 and aposymbiotic R. microsporus ATCC 62417/S) were used to visualize the presence or absence of endosymbiotic *M. rhizoxinica*. The fungal hyphae were stained with 5 μM Syto 9 (Invitrogen) for 5 to 10 min. Fluorescent microscopy was carried out using a spinning disc microscope (Axio Observer microscope-platform equipped with Cell Observer SD; Zeiss), and images were captured using Zeiss-Zen software.

### *P. aurantium* amoeba predation assay on *R. microsporus* spores.

R. microsporus was grown on PDA plates for 7 days. Spores were harvested using 12 mL of NaCl (0.15 M). The spore solution was centrifuged at 5,000 × *g* for 15 min. The resulting pellet was resuspended in 1 mL 50% glycerol, and spores were counted in a hemocytometer.

Trophozoites of *P. aurantium* were pregrown in petri dishes with PB with *R. mucilaginosa* as a food source. The dishes were washed with PB, and the adherent amoeba were harvested by scraping the surfaces of the dishes. Cell numbers were determined in a CASY cell counter (OMNI Life Science), and a total of 10^5^ amoeba cells were seeded in 24-well tissue culture plates containing 500 μL of PB. These amoeba cells were further confronted with the spores of R. microsporus directly (dormant spores) or after preincubation at 30°C for 3 h (swollen spores), in prey-predator ratios ranging from 0.1:1 to 10:1. After 24 h of coincubation, the numbers of amoebae were determined in a hemocytometer and compared to the fungus-free controls (prey predator ratio of 0:1 in Fig. 2B).

To visualize predation of *P. aurantium* on spores of R. microsporus, ~500 μL of spore suspension was mixed with fluorescein isothiocyanate (FITC) staining solution (1 mg/10 mL 0.1 M Na_2_CO_3_) and incubated at 37°C for 30 min and 8,000 rpm under light exclusion. After incubation, the spores were centrifuged and washed three times with PB to remove unbound staining. Stained spores were coincubated with *P. aurantium* trophozoites and visualized using a fluorescence spinning disc microscope (Axio Observer microscope-platform equipped with Cell Observer SD; Zeiss) with an excitation/emission range of 495/519 nm.

### Culture extraction and compound isolation.

Axenic *Mycetohabitans* cultures were grown in 400 mL of MGY medium containing 10% TSB (17 g/L tryptone, 3 g/L soy, 5 g/L NaCl, 2.5 g/L K_2_HPO_4_, 2.5 g/L glucose) in 1-L baffled Erlenmeyer flasks for 7 days at 30°C and 110 rpm. Fungal strains (ATCC 62417 and ATCC 62417/S) were grown on 20 PDA-containing standard-sized petri dishes at 30°C for 7 days. Both liquid bacterial cultures and fungal agar plates were exhaustively extracted with 400 mL of ethyl acetate. Extracts were concentrated on a rotary vacuum evaporator and then dried. Dry extracts were dissolved in 1 mL of methanol and analyzed by HPLC and HPLC-HRESI-MS as described previously (47). The identity of the rhizoxin derivatives was verified by comparison to authentic references (47) (see Fig. S2). After HPLC analysis, crude extracts were dried and dissolved in 1 mL of DMSO for bioactivity assays. The volume of DMSO was adjusted for the bacterial extracts, making all samples equal to a final OD_600_ of ~3.5. Rhizoxin concentration was calculated by integration of the peak areas at 310 nm with Shimadzu ClassVP software (version 6.14 SP1).

For the isolation of rhizoxin S2, *M. rhizoxinica* HKI-0454 was cultured in 5.6 L of MGY 10% TSB equally distributed across 1-L baffled Erlenmeyer flasks. Compounds were isolated as described previously (36, 47).

### *P. aurantium* plaque assay.

Amoeba cells were seeded in the 96-well plates (TC treated; Costar, Corning, NY) at a concentration of 10^5^ cells/mL in PB. Cells were either left untreated or incubated in the presence of 2% crude extract from bacterial or fungal cultures for 1 h or with pure bacterial rhizoxin S2 (final concentration, 0.1 to 5 μM) for 24h. Incubation with media or solvent (DMSO) was included as a control. Afterward, 20 μL of cell suspension was pipetted into the middle of a PB agar plate covered with a dense layer of *R. mucilaginosa* as a food source. The predation plaque, appearing as a halo due to the clearance of the yeast, was measured after 5 days. Each experiment was performed in three biological replicates.

### *P. aurantium* survival assay.

Amoeba cells were seeded in 24-well plates (Falcon, TC-treated; Corning), at a concentration of 10^6^ cells/mL in PB. Rhizoxin S2 was prepared in the working concentration of 100 μM. In a defined concentration range, rhizoxin S2 was added to the cells and filled up to final volume of 500 μL. Amoeba cells were further incubated in the presence of *R. mucilaginosa* for 24 h. The next day, the cells were scraped off the bottoms of the wells, and the number of viable cells was counted manually in an improved Neubauer counting chamber. Each experiment was performed in three biological replicates. A four-parameter sigmoidal concentration-dependent response curve was fitted using Prism version 6.03 (GraphPad Software, La Jolla, CA), and the IC_50_ and 95% CI values were determined.

### *C. elegans* feeding inhibition assay.

C. elegans was cultured on NGM OP50 agar plates at 20°C for 4 days. Nematodes were harvested by washing the agar plates with 12 mL of sterile K-medium (3.1 g/L NaCl, 2.4 g/L KCl). Worms were allowed to settle to the bottom through incubation at 4°C for 20 min. The supernatant was carefully removed, and the worms were washed with 12 mL of K-medium twice. On the last washing step, the supernatant was removed, and worms were resuspended in 5 mL of fresh K-medium.

Liquid OP50 cultures were grown in Lysogeny broth (10 g/L tryptone, 5 g/L yeast extract, 10 g/L NaCl [pH 7.0]) at 37°C overnight. Cells were harvested by centrifugation at 8,000 rpm for 5 min and resuspended in K-medium, and the OD_600_ was adjusted to 1.2. Individual wells of six-well cell culture plates (Costar, Corning) were seeded with 1.76 mL of Escherichia coli suspension (OD_600_ 1.2) and 200 μL of nematode suspension.

Crude culture extracts, dissolved in DMSO, were added to the wells (40 μL). DMSO served as a blank control, and 40 μL of 900 mM boric acid (final concentration of 18 mM) was used as a positive control. To determine the natural viability of E. coli OP50 cells, wells without nematodes were included in each assay. All plates were incubated for 7 days at 20°C and 90 rpm, and the OD_600_ was measured every 24 h. The number of viable nematode worms in the suspension is directly related to the E. coli cell density. Mean OD_600_ values from three independent experiments (*n* = 3 biological replicates) were plotted as a percentage of the starting OD_600_ ± one standard error of the mean (SEM).

For potency assessment, rhizoxin S2 was dissolved in DMSO and added (40 μL) to the wells with a final concentration range of 0.1 to 1,000 μM (three replicates per concentration). The OD_600_ values were measured as described above, and the IC_50_ and 95% CI values were calculated using GraphPad.

### *A. avenae-R. microsporus* coculture.

The nematode A. avenae (Bastian, 1865) was kindly provided by Markus Künzler (ETH Zürich, Switzerland). *A. avenae* was maintained on a nonsporulating strain of Botrytis cinerea (BC-3) growing on malt extract agar plates (MEA) supplemented with 100 μg/mL chloramphenicol at 20°C (63). For feeding assays, nematodes were harvested from cocultures by Baermann funneling overnight (64, 65). Nematodes were collected in 50-mL Falcon tubes, followed by incubation at 4°C for 2 h. The supernatant was removed, and worms were resuspended in 50 mL of sterile K-medium supplemented with 25 μM kanamycin and 100 μM Geneticin. The worm suspension was incubated at room temperature for 2 h to eliminate remaining fungal spores. Worms were washed with 50 mL of K-medium twice, resuspended in 5 mL of K-medium, and added (500 μL) to PDA plates containing 7-day-old cultures of symbiotic R. microsporus (ATCC 62417) and aposymbiotic R. microsporus (ATCC 62417/S) in triplicates. After incubation at 21°C for 3 weeks, worms were harvested from cocultures by Baermann funneling as described above. After antibiotic treatment and washing with K-medium, worms were transferred on to sterilization agar plates (15 g/L agar, 50 μg/mL kanamycin, 200 μM Geneticin) and incubated at 21°C overnight (66). Nematode movement was recorded in time-lapse videos (1 min) with a frame rate of 1 fps using a Zeiss Axio Zoom.V16 stereomicroscope (Zeiss, Oberkochen, Germany).

### Image analysis and mathematical quantification of *A. avenae* migration.

Transmitted-light time series images of free-moving worms were analyzed via an automated image processing and quantification algorithm. The raw images were provided in the native Carl-Zeiss image format “CZI,” whereas the processing was carried out in a novel graphical image analysis language JIPipe (www.jipipe.org), available as a plugin in ImageJ (v.1.53c). Images recorded at both 25× and 32× magnifications were used, without having to modify the analysis steps or controlling parameters. At these magnifications, individual worms were easily identifiable without fluorescence labeling, thus avoiding interference with biological function (67). The worm number in the microscope’s field of view was high enough to provide statistically meaningful quantification results. The step-by-step workflow is summarized in Fig. S3A, whereas images depicting representative intermediate results of the processing are presented in Fig. S3B. The images were first corrected for uneven transmitted light illumination, followed by Laplacian sharpening (3 × 3 pixels) using a Hessian filter with a 5-pixel kernel diameter (68). Here, the largest Hessian eigenvalues were used to build an image that showed the worms at enhanced contrast due to the continuously curved body outline of a typical nematode. The Hessian eigenimage was further processed to produce a high-fidelity segmentation of individual worms: a Gaussian blurring using a 3-pixel disk structural element was followed by thresholding using the Default algorithm (i.e., a modified IsoData algorithm) and morphological dilation (1 pixel) and hole filling. The nonworm image elements were removed by applying the Remove Outliers command at sizes below 50 pixels.

10.1128/mbio.01440-22.9 FIG S3 Automated workflow of worm liveliness analysis and illustrative steps of the image segmentation process. (A) The preprocessing and per-timeframe segmentation of images were carried out by using a custom-written JIPipe project (www.jipipe.org). The processing steps were as follows: illumination correction (with 20-pixel Gaussian filtering); Laplacian sharpening (3 × 3 pixels); Hessian filtering (5 pixels, largest eigenvalue); Gaussian filtering (3 pixels); thresholding (default method as provided by ImageJ); dilation (1 pixel); morphological hole filling; and remove outliers (50 pixels size threshold). The resulting images were tracked with JIPipe’s “Split into connected components” algorithm, producing both the per-worm segmented areas and the per-track combined area covered by each worm (the so-called worm footprint). The per-track worm footprints were divided by the per-worm areas at each timepoint, thus providing the so-called liveliness ratio (LR). (B) The images were processed according to the workflow (see Fig. S2A) described in detail in the Materials and Methods section “Image analysis and mathematical quantification of A. avenae migration.” The illustrated steps are as follows: the transmitted light raw image; the preprocessed image (after Gaussian filtering); and the binarized image (after remove outliers). Download FIG S3, TIF file, 1.0 MB. Copyright © 2022 Richter et al. 2022 Richter et al. https://creativecommons.org/licenses/by/4.0/ This content is distributed under the terms of the Creative Commons Attribution 4.0 International license.

The per-image segmentation of individual worms was followed by tracking using the connected components algorithm of JIPipe (“Split into connected components” node). The tracking workflow was carried out by two parallel branches: one algorithm extracted the per-worm and per-track information of individual worm areas, whereas the other branch calculated the total area covered by each worm while counting each worm-covered pixel only once. The latter workflow thus provided a footprint of a worm covered during the time-series experiment (see Fig. S4; see also Video S8 [https://doi.org/10.5281/zenodo.6827988]). In the next JIPipe compartment, the footprint of the worm was divided by the individual worm areas recorded at each time point; the resulting ratio was 1.0 for a fully immobile worm, whereas more active worms produced higher ratios. This ratio was termed the liveliness ratio (LR) because it quantifies the agility of a worm. The average ratio per track was calculated by taking the arithmetic mean and standard deviation of the per time point ratio values. The ratio varied with time due to variance of the apparent area of the worm as measured by the per-image segmentation algorithm. Tracks that were characterized by very high standard deviations were excluded from further analysis because a high standard deviation would indicate that the segmentation was unreliable at some time point(s) of the track, e.g., due to a merging of two or more worms into a cluster that could not be resolved by the segmentation algorithm. This ratio was called liveliness ratio (LR) because of its description of the agility of a worm. Nematodes with LR values below 1.4 were considered immobile, whereas a fast-moving worm would be characterized by a high LR value, with an average of 5.5 ± 0.5 over all experiments and conditions. The average and standard deviation LR results were saved in CSV file formats per experiment and condition for further analysis.

10.1128/mbio.01440-22.10 FIG S4 Images from a 39-frame movie showing the summarized outlines of all tracked worms. The yellow lines are the outlines of each tracked worm at every time point. The white elongated objects indicate the segmented worms. The segmentation and tracking were carried out as described in Fig. S3A. Of the 39-frame time series of images, the images for time (t) points 1, 10, 20, 30, and 39 are shown. The entire time series of the tracks and segmented worms can be viewed as animation in Video S5 (https://doi.org/10.5281/zenodo.6827988). Download FIG S4, TIF file, 1.2 MB. Copyright © 2022 Richter et al. 2022 Richter et al. https://creativecommons.org/licenses/by/4.0/ This content is distributed under the terms of the Creative Commons Attribution 4.0 International license.

An example of the time series of segmented worms and their tracks can be viewed in Video S9 (https://doi.org/10.5281/zenodo.6827988). Video S10 (https://doi.org/10.5281/zenodo.6827988) shows the velocity distribution of the same movie. The isotropic nature of the velocity vector-distribution points to a random walk-type worm movement in the movie.

### *A. avenae* feeding on *R. microsporus*.

To monitor feeding of *A. avenae* on R. microsporus, a 0.2-μm Luer μ-slide (Ibidi GmbH, Gräfelfing, Germany) was filled with sterile potato dextrose broth (Becton Dickinson). R. microsporus mycelium (ATCC 62417 or ATCC 62417/S) was added to one of the inoculation holes, and slides were incubated at 30°C for 2 days to allow hyphae to grow into the microchannel. Sterilized *A. avenae* (see above) was added to the second inoculation hole, and slides were incubated at 21°C overnight. Feeding of *A. avenae* on R. microsporus hyphae was observed using a Zeiss spinning disc microscope (Axio Observer microscope-platform equipped with Cell Observer SD; Zeiss).

### Exposure of *A. avenae* to rhizoxin S2.

A. avenae was maintained and harvested by Baermann funneling as described above. After incubation with 25 μM kanamycin and 100 μM Geneticin, nematodes were washed twice with 50 mL of K-medium and resuspended in 5 mL of K-medium. Individual wells of 24-well cell culture plates (Costar, Corning) were seeded with 440 μL of K-medium and 50 μL of nematode suspension. Pure rhizoxin S2, dissolved in 50% DMSO, was added to the wells (10 μL) with a final concentration range of 100 to 500 μM. DMSO (10 μL) served as blank control and 10 μL of 5.7 mM ivermectin (final concentration, 114 μM) was used as positive control. Each treatment was performed in three independent experiments (*n* = 3 biological replicates). Nematode movement was recorded in time-lapse videos (1 min) with a frame rate of 1 fps using a Zeiss Axio Zoom.V16 stereomicroscope (Zeiss).

### Statistical analysis.

Raw data from *P. aurantium*, C. elegans, or *A. avenae* survival experiments were processed in MS Excel and statistical analysis was performed in GraphPad Prism 6.03. An unpaired *t* test with Welch’s correction was used to study the following relationships: (i) the ingestion of swollen R. microsporus spores versus dormant spores by *P. aurantium* and (ii) the liveliness ratio of *A. avenae* feeding on either symbiotic R. microsporus or endosymbiont-free R. microsporus. To study the effect of fungal (symbiotic and apo-symbiotic R. microsporus) and bacterial (*Mycetohabitans* sp.) culture extracts on the survival of *P. aurantium* and C. elegans, one-way analysis of variance (ANOVA) was used in combination with the Tukey HSD test function. The Brown-Forsythe test was used to test for equal variance. For all statistical tests performed, *P* values of* *<0.05 were considered statistically significant.

### Alignment of β-tubulin proteins.

Amino acid sequences from β-tubulin genes of *Protostelium aurantium*, Dictyostelium discoideum, *Acanthamoebae castellanii*, Caenorhabditis elegans, and *Rhizopus oryzae* were downloaded from the Universal Protein Resource (https://www.uniprot.org/). β-Tubulin amino acid sequences from Rhizopus microsporus var. microsporus and *Bursaphelenchus okinawaensis* were downloaded from GenBank. Sequence alignments were carried using ClustalW (69). Alignments were generated using a gap open penalty of 10 and a gap extension penalty of 0.1, as implemented in the MEGA7 package (Molecular Evolutionary Genetics Analysis software, version 5.0) (70).

### Data availability.

All data generated or analyzed during this study are included in the manuscript and in the supporting files. Source data files have been provided for all videos (https://doi.org/10.5281/zenodo.6827988).
