# Supplementary material for: Toxin-Producing Endosymbionts Shield Pathogenic Fungus against Micropredators
Source: mBio. 2022 Aug 25;13(5):e01440-22. doi: 10.1128/mbio.01440-22 (PMC9600703; doi:10.1128/mbio.01440-22)
Supplement: TABLE S1 [file mbio.01440-22-s0005.docx]

**Table S1. (A)** Approximate probabilities (p) of unpaired *t*-test with Welch’s correction for the mean diameter of dormant and swollen spores (in μm) of *Rhizopus microsporus* ATCC62417. **(B)** Approximate probabilities (p) of Brown-Forsythe test, one-way analysis of variance (ANOVA), and Tukey HSD Post Hoc test for the survival of *Protostelium aurantium* following exposure to dormant and swollen spores of *R. microsporus* ATCC62417. Homogeneous data (non-significant Brown-Forsythe) is shown in black numbers and non-homogeneous data (significant Brown-Forsythe) is highlighted in red numbers. P-values with *p<0.05* were considered statistically significant (highlighted in grey).

**A**

| **Unpaired *t*-test with Welch's correction** |  |
| --- | --- |
| P value | 0.0081 |
| P value summary | ** |
| Significantly different (*p<0.05*)? | Yes |
| One- or two-tailed P value? | Two-tailed |
| Welch-corrected *t*, df | *t* = 5.933, df = 3.185 |
| **How big is the difference?** |  |
| Mean of column A | 5.23 |
| Mean of column B | 9.02 |
| Difference between means (B - A) ± SEM | 3.790 ± 0.6388 |
| 95% confidence interval | 1.822 to 5.758 |
| R squared (eta squared) | 0.917 |
| **F test to compare variances** |  |
| F, DFn, Dfd | 3.047, 2, 2 |
| P value | 0.4942 |
| P value summary | ns |
| Significantly different (*p<0.05*)? | No |

**B**

| **Brown-Forsythe test** |  |
| --- | --- |
| F (DFn, DFd) | 0.7100 (7, 16) |
| P value | 0.6646 |
| P value summary | ns |
| Are SDs significantly different (*p<0.05*)? | No |

| **ANOVA Summary** |  |
| --- | --- |
| F | 9.462 |
| P value | 0.0001 |
| P value summary | *** |
| Significant diff. among means (*p<0.05*)? | Yes |
| R square | 0.8054 |

| **ANOVA Table** | **SS** | **DF** | **MS** | **F (DFn, DFd)** | **P value** |
| --- | --- | --- | --- | --- | --- |
| Treatment (between columns) | 1.21E+11 | 7 | 1.72E+10 | F (7, 16) = 9.462 | P=0.0001 |
| Residual (within columns) | 2.91E+10 | 16 | 1.82E+09 |  |  |
| Total | 1.50E+11 | 23 |  | $-$ |  |

| **Prey-predator ratio** | | | **Mean Diff.** | **95% CI** |  |  | **Significant?** | **Summary** |
| --- | --- | --- | --- | --- | --- | --- | --- | --- |
| **Dormant spores** | | |  |  |  |  |  |  |
| 0 | vs. | 0.1 | 2.33E+04 | $-$9.73E+04 | to | 1.44E+05 | No | ns |
| 0 | vs. | 1 | 7.00E+04 | $-$5.06E+04 | to | 1.91E+05 | No | ns |
| 0 | vs. | 10 | 1.97E+05 | 7.60E+04 | to | 3.17E+05 | Yes | *** |
| 0.1 | vs. | 1 | 4.67E+04 | $-$7.40E+04 | to | 1.67E+05 | No | ns |
| 0.1 | vs. | 10 | 1.73E+05 | 5.27E+04 | to | 2.94E+05 | Yes | ** |
| 1 | vs. | 10 | 1.27E+05 | 6.04E+03 | to | 2.47E+05 | Yes | * |
|  | | |  |  |  |  |  |  |
| **Swollen spores** | | |  |  |  |  |  |  |
| 0 | vs. | 0.1 | -2.67E+04 | $-$1.47E+05 | to | 9.40E+04 | No | ns |
| 0 | vs. | 1 | 4.67E+04 | $-$7.40E+04 | to | 1.67E+05 | No | ns |
| 0 | vs. | 10 | 1.30E+05 | 9.38E+03 | to | 2.51E+05 | Yes | * |
| 0.1 | vs. | 1 | 7.33E+04 | $-$4.73E+04 | to | 1.94E+05 | No | ns |
| 0.1 | vs. | 10 | 1.57E+05 | 3.60E+04 | to | 2.77E+05 | Yes | ** |
| 1 | vs. | 10 | 8.33E+04 | $-$3.73E+04 | to | 2.04E+05 | No | ns |

ns: not significant, **p<0.0332*, ***p<0.0021*, ****p<0.0002*, *****p<0.0001*.
