# Supplementary material for: Toxin-Producing Endosymbionts Shield Pathogenic Fungus against Micropredators
Source: mBio. 2022 Aug 25;13(5):e01440-22. doi: 10.1128/mbio.01440-22 (PMC9600703; doi:10.1128/mbio.01440-22)
Supplement: TABLE S2 [file mbio.01440-22-s0006.docx]

**Table S2.** Approximate probabilities (p) of Brown-Forsythe test, one-way analysis of variance (ANOVA), and Tukey HSD Post Hoc test for the survival of *Protostelium aurantium* following exposure to 2% crude culture extract from symbiotic *Rhizopus* *microsporus* (RMsym), endosymbiont-free *Rhizopus* *microsporus* (RMapo), or solvent control (DMSO). Homogeneous data (non-significant Brown-Forsythe) is shown in black numbers and non-homogeneous data (significant Brown-Forsythe) is highlighted in red numbers. P-values with *p<0.05* were considered statistically significant (highlighted in grey).

| **Brown-Forsythe test** |  |
| --- | --- |
| F (DFn, DFd) | 1.245 (13, 28) |
| P value | *p = 0.3016* |
| P value summary | ns |
| Are SDs significantly different (*p<0.05*)? | No |

| **ANOVA Summary** |  |
| --- | --- |
| F | 320.1 |
| P value | *p<0.0001* |
| P value summary | **** |
| Significant diff. among means (*p<0.05*)? | Yes |
| R square | 0.9933 |

| **ANOVA Table** | **SS** | **DF** | **MS** | **F (DFn, DFd)** | **P value** |
| --- | --- | --- | --- | --- | --- |
| Treatment (between columns) | 4656 | 13 | 358.2 | F (13, 28) = 320.1 | *p<0.0001* |
| Residual (within columns) | 31.33 | 28 | 1.119 |  |  |
| Total | 4688 | 41 |  |  |  |

| **Strain comparison** | | | **Mean Diff.** | **95% CI** | ***p<0.0001*?** | **Summary** |
| --- | --- | --- | --- | --- | --- | --- |
| RMsym | vs. | DMSO | $-$15.33 | $-$18.49 to $-$12.17 | Yes | **** |
| RMapo | vs. | DMSO | $-$1.667 | $-$4.828 to 1.495 | No | ns |
| RMsym | vs. | RMapo | 13.67 | 10.51 to 16.83 | Yes | **** |

ns: not significant, **p<0.0332*, ***p<0.0021*, ****p<0.0002*, *****p<0.0001*.
