# Supplementary material for: Toxin-Producing Endosymbionts Shield Pathogenic Fungus against Micropredators
Source: mBio. 2022 Aug 25;13(5):e01440-22. doi: 10.1128/mbio.01440-22 (PMC9600703; doi:10.1128/mbio.01440-22)
Supplement: TABLE S3 [file mbio.01440-22-s0007.docx]

**Table S3.** Approximate probabilities (p) of Brown-Forsythe test, one-way analysis of variance (ANOVA), and Tukey HSD Post Hoc test for the survival of *Protostelium aurantium* following exposure to 2% crude extract from axenic endosymbiotic *Mycetohabitans rhizoxinica* HKI-0454 (Mr), *Mycetohabitans endofungorum* HKI-0456 (Me), rhizoxin-deficient mutant (*ΔrhiG*) cultures, solvent control (DMSO), and untreated control. Homogeneous data (non-significant Brown-Forsythe) is shown in black numbers and non-homogeneous data (significant Brown-Forsythe) is highlighted in red numbers. P-values with *p<0.0001* were considered statistically significant (highlighted in grey).

| **Brown-Forsythe test** |  |
| --- | --- |
| F (DFn, DFd) | 0.7619 (7, 16) |
| P value | *p = 0.6264* |
| P value summary | ns |
| Are SDs significantly different (*p<0.05*)? | No |

| **ANOVA Summary** |  |
| --- | --- |
| F | 212.8 |
| P value | *p<0.0001* |
| P value summary | **** |
| Significant diff. among means (*p<0.05*)? | Yes |
| R square | 0.9894 |

| **ANOVA Table** | **SS** | **DF** | **MS** | **F (DFn, DFd)** | **P value** |
| --- | --- | --- | --- | --- | --- |
| Treatment (between columns) | 2483 | 7 | 354.7 | F (7, 16) = 212.8 | *p<0.0001* |
| Residual (within columns) | 26.67 | 16 | 1.667 |  |  |
| Total | 2509 | 23 |  |  |  |

| **Strain Comparison** | | **Mean Diff.** | **95% CI** | ***p<0.05*?** | **Summary** |
| --- | --- | --- | --- | --- | --- |
| MR vs. | ME | 0 | $-$3.649 to 3.649 | No | ns |
|  | Δ*rhiG* | $-$23.33 | $-$26.98 to $-$19.68 | Yes | **** |
|  | Medium | $-$23 | $-$26.65 to $-$19.35 | Yes | **** |
|  | DMSO | $-$21.33 | $-$24.98 to $-$17.68 | Yes | **** |
|  | Untreated control | $-$7.667 | $-$11.32 to $-$4.017 | Yes | **** |
| ME vs. | Δ*rhiG* | $-$23 | $-$26.65 to $-$19.35 | Yes | **** |
|  | Medium | $-$24.33 | $-$27.98 to $-$20.68 | Yes | **** |
|  | DMSO | $-$23.33 | $-$26.98 to $-$19.68 | Yes | **** |
|  | Untreated control | $-$23 | $-$26.65 to $-$19.35 | Yes | **** |
| Δ*rhiG* vs. | Medium | $-$21.33 | $-$24.98 to $-$17.68 | Yes | **** |
|  | DMSO | $-$7.667 | $-$11.32 to $-$4.017 | Yes | **** |
|  | Untreated control | $-$23 | $-$26.65 to $-$19.35 | Yes | **** |
| Medium vs. | DMSO | $-$24.33 | $-$27.98 to $-$20.68 | Yes | **** |
|  | Untreated control | 0.3333 | $-$3.316 to 3.983 | No | ns |
| DMSO vs. | Untreated control | 2 | $-$1.649 to 5.649 | No | ns |

ns: not significant, **p<0.0332*, ***p<0.0021*, ****p<0.0002*, *****p<0.0001*.
