# Supplementary material for: Toxin-Producing Endosymbionts Shield Pathogenic Fungus against Micropredators
Source: mBio. 2022 Aug 25;13(5):e01440-22. doi: 10.1128/mbio.01440-22 (PMC9600703; doi:10.1128/mbio.01440-22)
Supplement: TABLE S4 [file mbio.01440-22-s0008.docx]

**Table S4.** Approximate probabilities (p) of Brown-Forsythe test, one-way analysis of variance (ANOVA), and Tukey HSD Post Hoc test for the survival of *Caenorhabditis elegans* following exposure to 2% crude culture extract from symbiotic *Rhizopus* *microsporus* (RMsym), endosymbiont-free *Rhizopus* *microsporus* (RMapo), axenic endosymbiotic *Mycetohabitans rhizoxinica* HKI-0454 (ME), *Mycetohabitans endofungorum* HKI-0456 (ME), rhizoxin-deficient mutant (*ΔrhiG*) cultures, and various concentrations of pure rhizoxin S2 (rhi S2). The following controls were included in each experiment: extract of culture medium (Control), solvent control (DMSO), reactions not containing *C. elegans* ($-$ N2WT), and boric acid as positive control. Homogeneous data (non-significant Brown-Forsythe) is shown in black numbers and non-homogeneous data (significant Brown-Forsythe) is highlighted in red numbers. P-values with *p<0.05* were considered statistically significant (highlighted in grey).

| **Brown-Forsythe test** |  |
| --- | --- |
| F (DFn, DFd) | 0.7427 (11, 24) |
| P value | 0.6892 |
| P value summary | ns |
| Are SDs significantly different (*p<0.05*)? | No |

| **ANOVA Summary** |  |
| --- | --- |
| F | 99.61 |
| P value | <0.0001 |
| P value summary | **** |
| Significant diff. among means (*p<0.05*)? | Yes |
| R square | 0.9786 |

| **ANOVA Table** | **SS** | **DF** | **MS** | **F (DFn, DFd)** | **P value** |
| --- | --- | --- | --- | --- | --- |
| Treatment (between columns) | 24113 | 11 | 2192 | F (11, 24) = 99.61 | P<0.0001 |
| Residual (within columns) | 528.2 | 24 | 22.01 |  |  |
| Total | 24641 | 35 |  |  |  |

| **Strain Comparison** | | **Mean Diff.** | **95% CI** | ***p<0.05*?** | **Summary** |
| --- | --- | --- | --- | --- | --- |
| Boric acid vs. | DMSO | 59 | 46.00 to 72.00 | Yes | **** |
|  | RMsym | 41.67 | 28.66 to 54.67 | Yes | **** |
|  | RMapo | 65 | 52.00 to 78.00 | Yes | **** |
|  | MR | 56 | 43.00 to 69.00 | Yes | **** |
|  | ME | 52.33 | 39.33 to 65.34 | Yes | **** |
|  | Δ*rhiG* | 69.93 | 56.93 to 82.94 | Yes | **** |
|  | 1 µM rhi S2 | 65.67 | 52.66 to 78.67 | Yes | **** |
|  | 250 µM rhi S2 | 48 | 35.00 to 61.00 | Yes | **** |
|  | 1000 µM rhi S2 | 29.33 | 16.33 to 42.34 | Yes | **** |
|  | Control | 66 | 53.00 to 79.00 | Yes | **** |
|  | $-$ N2WT | $-$13.33 | $-$26.34 to $-$0.3308 | Yes | * |
| DMSO vs. | RMsym | $-$17.33 | $-$30.34 to $-$4.331 | Yes | ** |
|  | RMapo | 6 | $-$7.003 to 19.00 | No | ns |
|  | MR | $-$3 | $-$16.00 to 10.00 | No | ns |
|  | ME | $-$6.667 | $-$19.67 to 6.336 | No | ns |
|  | Δ*rhiG* | 10.93 | $-$2.069 to 23.94 | No | ns |
|  | 1 µM rhi S2 | 6.667 | $-$6.336 to 19.67 | No | ns |
|  | 250 µM rhi S2 | $-$11 | $-$24.00 to 2.003 | No | ns |
|  | 1000 µM rhi S2 | $-$29.67 | $-$42.67 to $-$16.66 | Yes | **** |
|  | Control | 7 | $-$6.003 to 20.00 | No | ns |
|  | $-$ N2WT | $-$72.33 | $-$85.34 to $-$59.33 | Yes | **** |
| RMsym vs. | RMapo | 23.33 | 10.33 to 36.34 | Yes | **** |
|  | MR | 14.33 | 1.331 to 27.34 | Yes | * |
|  | ME | 10.67 | $-$2.336 to 23.67 | No | ns |
|  | Δ*rhiG* | 28.27 | 15.26 to 41.27 | Yes | **** |
|  | 1 µM rhi S2 | 24 | 11.00 to 37.00 | Yes | **** |
|  | 250 µM rhi S2 | 6.333 | $-$6.669 to 19.34 | No | ns |
|  | 1000 µM rhi S2 | $-$12.33 | $-$25.34 to 0.6692 | No | ns |
|  | Control | 24.33 | 11.33 to 37.34 | Yes | **** |
|  | $-$ N2WT | $-$55 | $-$68.00 to $-$42.00 | Yes | **** |
| RMapo vs. | MR | $-$9 | $-$22.00 to 4.003 | No | ns |
|  | ME | $-$12.67 | $-$25.67 to 0.3358 | No | ns |
|  | Δ*rhiG* | 4.933 | $-$8.069 to 17.94 | No | ns |
|  | 1 µM rhi S2 | 0.6667 | $-$12.34 to 13.67 | No | ns |
|  | 250 µM rhi S2 | $-$17 | $-$30.00 to $-$3.997 | Yes | ** |
|  | 1000 µM rhi S2 | $-$35.67 | $-$48.67 to $-$22.66 | Yes | **** |
|  | Control | 1 | $-$12.00 to 14.00 | No | ns |
|  | $-$ N2WT | $-$78.33 | $-$91.34 to $-$65.33 | Yes | **** |
| MR vs. | ME | $-$3.667 | $-$16.67 to 9.336 | No | ns |
|  | Δ*rhiG* | 13.93 | 0.9308 to 26.94 | Yes | * |
|  | 1 µM rhi S2 | 9.667 | $-$3.336 to 22.67 | No | ns |
|  | 250 µM rhi S2 | $-$8 | $-$21.00 to 5.003 | No | ns |
|  | 1000 µM rhi S2 | $-$26.67 | $-$39.67 to $-$13.66 | Yes | **** |
|  | Control | 10 | $-$3.003 to 23.00 | No | ns |
|  | $-$ N2WT | $-$69.33 | $-$82.34 to $-$56.33 | Yes | **** |
| ME vs. | Δ*rhiG* | 17.6 | 4.597 to 30.60 | Yes | ** |
|  | 1 µM rhi S2 | 13.33 | 0.3308 to 26.34 | Yes | * |
|  | 250 µM rhi S2 | $-$4.333 | $-$17.34 to 8.669 | No | ns |
|  | 1000 µM rhi S2 | $-$23 | $-$36.00 to $-$9.997 | Yes | **** |
|  | Control | 13.67 | 0.6642 to 26.67 | Yes | * |
|  | $-$ N2WT | $-$65.67 | $-$78.67 to $-$52.66 | Yes | **** |
| Δ*rhiG* vs. | 1 µM rhi S2 | $-$4.267 | $-$17.27 to 8.736 | No | ns |
|  | 250 µM rhi S2 | $-$21.93 | $-$34.94 to $-$8.931 | Yes | *** |
|  | 1000 µM rhi S2 | $-$40.6 | $-$53.60 to $-$27.60 | Yes | **** |
|  | Control | $-$3.933 | $-$16.94 to 9.069 | No | ns |
|  | $-$ N2WT | $-$83.27 | $-$96.27 to $-$70.26 | Yes | **** |
| 1 µM rhi S2 vs. | 250 µM rhi S2 | $-$17.67 | $-$30.67 to $-$4.664 | Yes | ** |
|  | 1000 µM rhi S2 | $-$36.33 | $-$49.34 to $-$23.33 | Yes | **** |
|  | Control | 0.3333 | $-$12.67 to 13.34 | No | ns |
|  | $-$ N2WT | $-$79 | $-$92.00 to $-$66.00 | Yes | **** |
| 250 µM rhi S2 vs. | 1000 µM rhi S2 | $-$18.67 | $-$31.67 to $-$5.664 | Yes | ** |
|  | Control | 18 | 4.997 to 31.00 | Yes | ** |
|  | $-$ N2WT | $-$61.33 | $-$74.34 to $-$48.33 | Yes | **** |
| 1000 µM rhi S2 vs. | Control | 36.67 | 23.66 to 49.67 | Yes | **** |
|  | $-$ N2WT | $-$42.67 | $-$55.67 to $-$29.66 | Yes | **** |
| Control vs. | $-$ N2WT | $-$79.33 | $-$92.34 to $-$66.33 | Yes | **** |

ns: not significant, **p<0.0332*, ***p<0.0021*, ****p<0.0002*, *****p<0.0001*.
