# Supplementary material for: Toxin-Producing Endosymbionts Shield Pathogenic Fungus against Micropredators
Source: mBio. 2022 Aug 25;13(5):e01440-22. doi: 10.1128/mbio.01440-22 (PMC9600703; doi:10.1128/mbio.01440-22)
Supplement: TABLE S5 [file mbio.01440-22-s0009.docx]

**Table S5.** Approximate probabilities (p) of unpaired *t*-test with Welch’s correction for the survival (liveliness ratio) of *Aphelenchus avenae* grazing on endosymbiont-free *Rhizopus microsporus* (column A) or symbiotic *R. microsporus* (column B).

| **Unpaired *t*-test with Welch's correction** |  |
| --- | --- |
| P value | *p = 0.0202* |
| P value summary | * |
| Significantly different (*p<0.05*)? | Yes |
| One- or two-tailed P value? | Two-tailed |
| Welch-corrected *t*, df | *t* = 3.760, df = 3.954 |
| **How big is the difference?** |  |
| Mean of column A | 6.248 |
| Mean of column B | 3.968 |
| Difference between means (B - A) ± SEM | –2.280 ± 0.6064 |
| 95% confidence interval | –3.972 to –0.5888 |
| R squared (eta squared) | 0.7815 |
| **F test to compare variances** |  |
| F, DFn, Dfd | 1.243, 2, 2 |
| P value | *p = 0.8917* |
| P value summary | ns |
| Significantly different (*p<0.05*)? | No |
